# Deciphering Immune-Associated Genes to Predict Survival in Clear Cell Renal Cell Cancer

**DOI:** 10.1155/2019/2506843

**Published:** 2019-12-07

**Authors:** Daixing Hu, Mi Zhou, Xin Zhu

**Affiliations:** ^1^Department of Urology, The First Affiliated Hospital of Chongqing Medical University, Chongqing, China; ^2^Department of Respiratory and Critical Care Medicine, The First Affiliated Hospital of Chongqing Medical University, Chongqing, China

## Abstract

**Background:**

To elucidate the correlations between tumor microenvironment and clinical characteristics as well as prognosis in clear cell renal cell cancer (ccRCC) and investigate the immune-associated genes by a comprehensive analysis of The Cancer Genome Atlas (TCGA) database.

**Methods:**

We collected mRNA expression profiles of 537 ccRCC samples from the TCGA database. Immune scores and stromal scores were calculated by applying the ESTIMATE algorithm. We evaluated the correlation between immune/stromal scores and clinical characteristics as well as prognosis. The differentially expressed genes (DEGs) were screened between high immune/stromal score and low immune/stromal score groups by the cutoff of |log (fold change)| > 1, *P* value <0.05 by using package “limma” in R. Functional enrichment analysis was performed by DAVID, and the protein-protein interaction network of intersected DEGs between stromal score and immune score groups was conducted using the STRING database. The Kaplan–Meier method was used to explore DEGs with predictive values in overall survival, and the prognostic DEGs were further validated in a Gene Expression Omnibus (GEO) dataset GSE29609.

**Results:**

A higher immune score was associated with T3/4 (vs. T1/2, *P* < 0.001), N1 (vs. N0, *P*=0.05), M1 (vs. M0, *P*=0.004), G3/4 (vs. G1/2, *P* < 0.001), advanced AJCC stage (*P* < 0.001), and shorter overall survival (*P*=0.04). Intersected DEGs between immune and stromal score groups were 48 upregulated and 47 downregulated genes, with 43 DEGs associated with overall survival in ccRCC. After validation by a cohort of 39 ccRCC cases with detailed follow-up information from GSE29609, six immune-associated DEGs including CASP5, HSD11B1, VSIG4, HMGCS2, HSD11B2, and OGDHL were demonstrated to be predictive of prognosis in ccRCC.

**Conclusions:**

Our study elucidated tight associations between immune score and clinical characteristics as well as prognosis in ccRCC. Moreover, six DEGs were explored and validated to exert predictive values in overall survival of ccRCC.

## 1. Background

Renal cell cancer (RCC), the common urological malignancy, was statistically predicted for 73,820 estimated new cases and 14,770 estimated deaths in 2019 in the United States [1]. Although detailed and various histopathological classifications were recommended by World Health Organization [2], clear cell renal cell cancer (ccRCC), papillary renal cell cancer (pRCC), and chromophobe renal cell cancer (chRCC) still remain the major histological types, with ccRCC accounting for the largest proportion. Radical surgical resection is the most effective therapy for RCC; however, approximately 30–40% of cases with ccRCC eventually progress into metastatic status postoperatively [3, 4]. Although systemic adjuvant therapy received limited effects on prognosis of advanced ccRCC, nivolumab, as a programmed death 1 (PD-1) immune checkpoint inhibitor antibody, demonstrated promising improved survival outcome in the metastatic ccRCC [5, 6]. Further investigation into the immune microenvironment of ccRCC is expected to reveal the underlying molecular mechanism and prognostic implications.

The Cancer Genome Atlas (TCGA) provided comprehensive genomic profiles and detailed clinical as well as follow-up information, making it suitable to investigate the correlation between immune and genomic features [7]. To evaluate the role of infiltrating stromal and immune cells in cancer biology, an algorithm called Estimation of STromal and Immune cells in MAlignant Tumours using Expression data (ESTIMATE) was raised and triggered a few genomic investigations on tumor-associated normal cells [8].

This study aimed to elucidate the correlations between tumor microenvironment and clinical characteristics as well as prognosis in ccRCC and investigate the prognosis-associated genes by a comprehensive exploration of the TCGA database and validation analysis based on the Gene Expression Omnibus (GEO) database.

## 2. Methods

Gene expression profile and clinical data of 537 ccRCC patients were downloaded from the TCGA database (https://tcga-data.nci.nih.gov/tcga/). Immune and stromal scores were calculated by using the ESTIMATE algorithm. Data analyses of differentially expressed genes (DEGs) were performed using package “limma” with threshold set as log FC (fold change) > 1 and *P* value <0.05 [9]. Heatmaps were performed using package “pheatmap.” Functional enrichment analyses of DEGs were performed by package “clusterProfiler,” “org.Hs.eg.db,” “enrichplot,” and “ggplot2.” Gene ontology (GO) categories consisted of biological processes (BPs) and molecular functions (MFs). KEGG (Kyoto Encyclopedia of Genes and Genomes) enrichment analysis was preformed to obtain significant associated pathways of DEGs [10]. The protein-protein interaction (PPI) network of intersected DEGs between stromal score and immune score groups was conducted using the STRING (https://string-db.org) [11]. The Kaplan–Meier method was used to explore DEGs with predictive values in overall survival, and the prognostic DEGs were further validated in a GEO dataset GSE29609. All statistical tests were done with R version 3.5 and GraphPad 7. A *P* < 0.05 was considered as a statistically significant difference.

## 3. Results

### 3.1. Correlation between Immune/Stromal Scores and Clinical Characteristics

A total of 537 ccRCC patients with gene expression profiles and clinical information were collected from the TCGA database. The patients were diagnosed as ccRCC between 1998 and 2013. Among them, 346 cases were male and 191 were female. According to the ESTIMATE algorithm, immune scores were distributed between −693.96 and 3328.21, while stromal scores ranged from −1433.77 to 1411.35.

**Figure 1:**
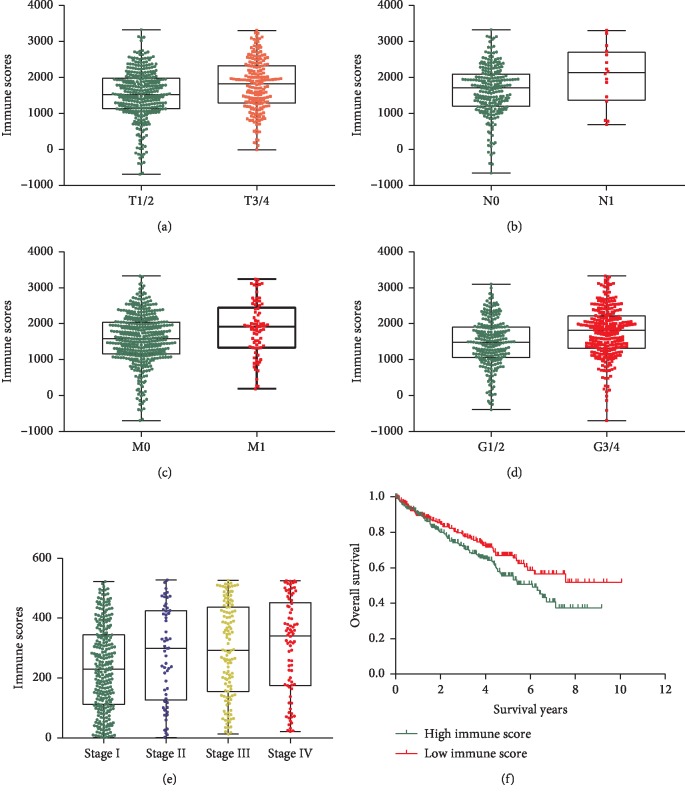
Immune scores were associated with clinical characteristics and overall survival. (a) Distribution of immune scores stratified by T status and box plot indicates a higher immune score was associated with T3/4 (vs. T1/2, *P* < 0.001). (b) Box plot indicated a higher immune score was associated with N1 (vs. N0, *P*=0.05). (c) Box plot indicates a higher immune score was associated with M1 (vs. M0, *P*=0.004). (d) Box plot indicated a higher immune score was associated with G3/4 (vs. G1/2, *P* < 0.001). (e) Box plot indicated a higher immune score was associated with the advanced AJCC stage (*P* < 0.001). (f) Kaplan–Meier survival curves for overall survival of ccRCC stratified by immune scores, indicating that the low-score group harbors survival advantage over the high immune score group (*P*=0.04).

To evaluate the correlations between clinical characteristics and immune or stromal scores, we compared and plotted the distribution of immune scores and stromal scores stratified by the T status, N status, M status, Fuhrman grade, and AJCC stage. We found that a higher immune score was associated with T3/4 (vs. T1/2, Figure 1(a), *P* < 0.001), N1 (vs. N0, Figure 1(b), *P*=0.05), M1 (vs. M0, Figure 1(c), *P*=0.004), and G3/4 (vs. G1/2, Figure 1(d), *P* < 0.001). With regard to the AJCC stage, the median average immune scores of stage IV ranked the highest of all stage classifications, while stage I harbored the lowest immune scores, indicating that higher immune scores predicted the advanced AJCC stage with statistical significance (Figure 1(e), *P* < 0.001). However, high stromal scores were only associated with T3/4 (vs. T1/2, Supplementary [Supplementary-material supplementary-material-1], *P*=0.03), without evidence to support significant correlation between stromal scores and N status, M status, Fuhrman grade, as well as AJCC stage (Supplementary Figures [Supplementary-material supplementary-material-1]–[Supplementary-material supplementary-material-1], *P* > 0.05).

To explore the potential correlations between immune scores and/or stromal scores with overall survival (OS), we retrieved 530 ccRCC cases with detailed follow-up information and divided them into high and low immune or stromal score groups. Kaplan–Meier survival curves (Figure 1(f)) showed that OS of cases with the low-score group of immune scores is longer than the cases in the high immune score group (Figure 1(f), *P*=0.04). However, cases with lower stromal scores did not show OS advantage over patients with higher stromal scores (Supplementary [Supplementary-material supplementary-material-1], *P*=0.22).

### 3.2. DEGs Associated with Tumor Microenvironment in ccRCC

**Figure 2:**
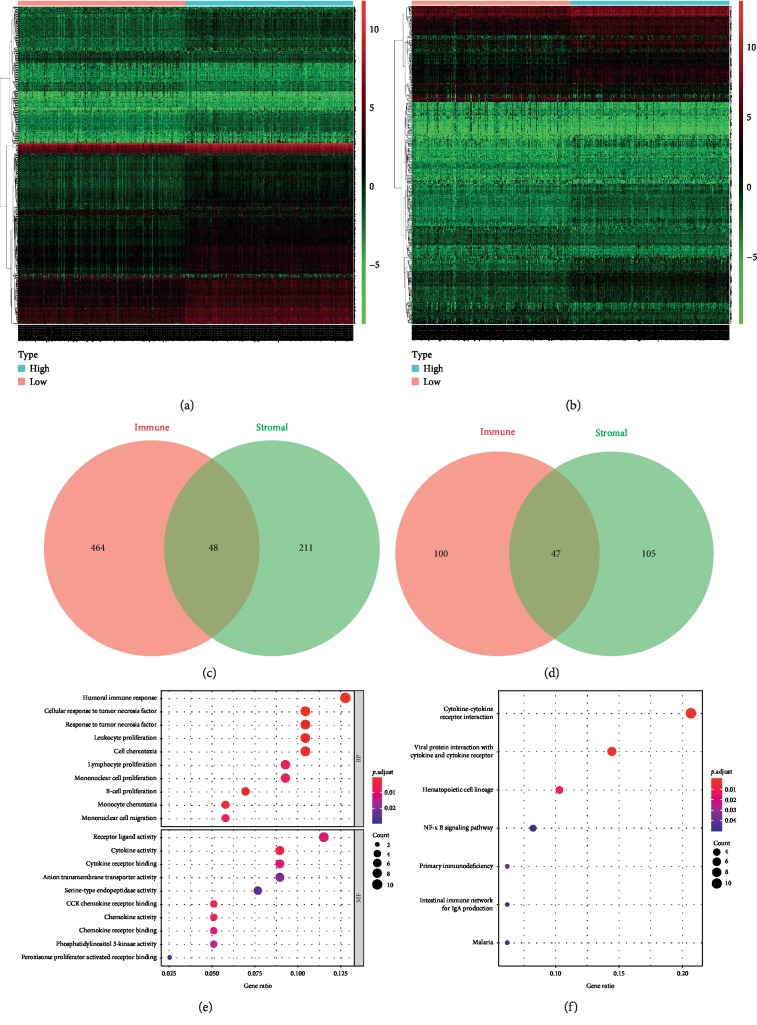
Exploration of DEGs associated with tumor microenvironment in ccRCC. (a) Heatmap of gene expression profiles of high immune score group and low immune score, log FC > 1, *P* < 0.05. (b) Heatmap of gene expression profiles of high stromal score group and low stromal score group, log FC > 1, *P* < 0.05. (c, d) Venn diagrams showed the intersect upregulated (c) or downregulated (d) DEGs between immune and stromal score groups. (e) The top 10 DEG-associated biological functions and molecular functions. (f) The potential pathways obtained by using KEGG analysis.

To investigate the DEGs associated with tumor microenvironment in ccRCC, we compared the gene expression profiles of cases stratified by high vs. low immune scores and/or stromal scores. The heatmaps of DEGs based on immune scores (Figure 2(a)) and stromal scores (Figure 2(b)) in ccRCC were presented for different expressions of genes in the separated specimen. With respect to immune scores, 512 genes were upregulated, while 147 genes were downregulated in the high-score group, compared with the low-score group (log FC > 1, *P* < 0.05). Moreover, comparison between the high and low stromal score groups showed 259 upregulated genes and 152 downregulated genes (log FC > 1, *P* < 0.05). Intersect genes between stromal score and immune score groups were achieved by Venn diagrams, with 48 intersective upregulated genes and 47 intersective downregulated genes (Figures 2(c) and 2(d)).

### 3.3. GO and Pathway Analysis of DEGs

**Figure 3:**
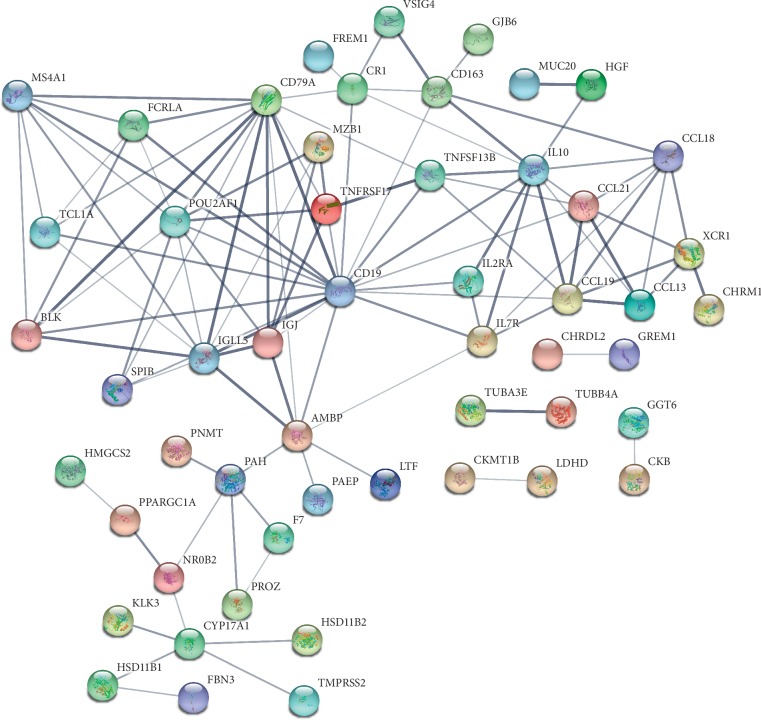
Protein-protein interaction (PPI) network of the DEGs in ccRCC.

To illustrate the functional implications of DEGs, the intersective DEGs were selected to evaluate biological functions and molecular functions by DAVID 6.8. The top 10 DEG-associated biological functions included humoral immune response, cellular response to tumor necrosis factor, response to tumor necrosis factor, leukocyte proliferation, cell chemotaxis, lymphocyte proliferation, mononuclear cell proliferation, B-cell proliferation, monocyte chemotaxis, and mononuclear cell migration, as demonstrated in Figure 2(e). Moreover, Figure 2(e) also showed the top 10 molecular functions including receptor ligand activity, cytokine activity, cytokine receptor binding, anion transmembrane transporter activity, serine-type endopeptidase activity, CCR chemokine receptor binding, chemokine activity, chemokine receptor binding, phosphatidylinositol 3-kinase activity, and peroxisome proliferator-activated receptor binding. The potential pathways involved were discovered by using KEGG analysis. These DEGs were enriched in cancer-related pathways including cytokine-cytokine receptor interaction, NF-kappa B signaling pathway, primary immunodeficiency, and intestinal immune network for IgA production (Figure 2(f)). The DEGs were also applied to construct a PPI network with the String database, as demonstrated in Figure 3.

### 3.4. Identification and Validation of Prognosis-Associated DEGs

**Figure 4:**
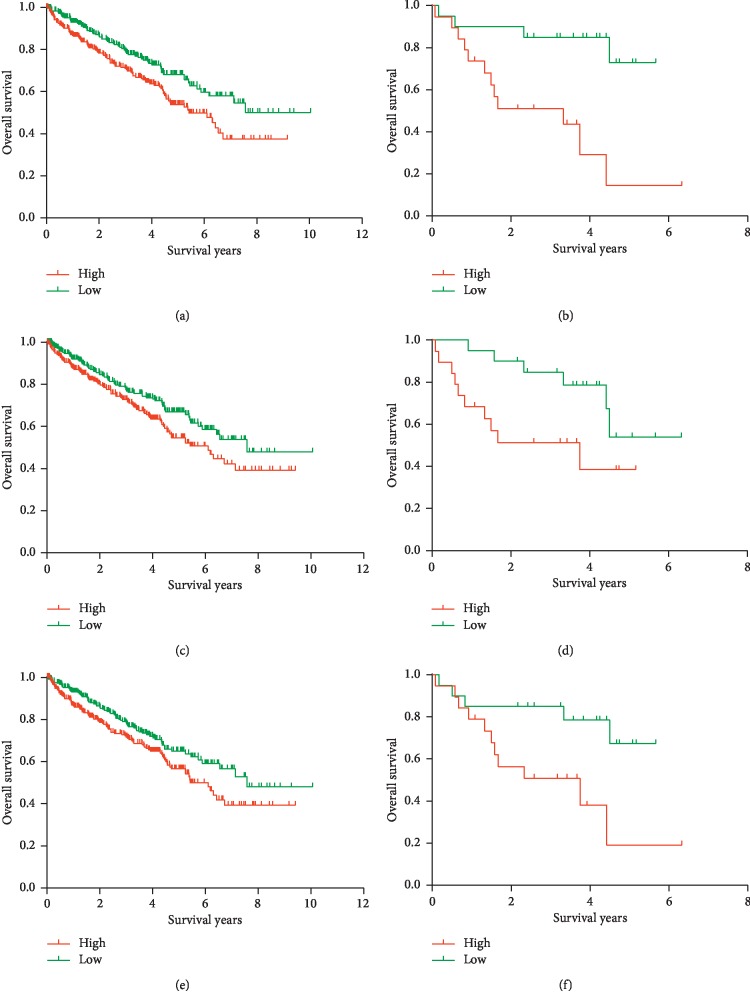
Validation of three upregulated DEGs from the TCGA database in the GEO database: Kaplan–Meier survival curve for (a) CASP5 in the TCGA database (*P*=0.005), (b) CASP5 in the GEO database (*P*=0.002), (c) HSD11B1 in the TCGA database (*P*=0.003), (d) HSD11B1 in the GEO database (*P*=0.05), (e) VSIG4 in the TCGA database (*P*=0.03), and (f) VSIG4 in the GEO database (*P*=0.05).

**Figure 5:**
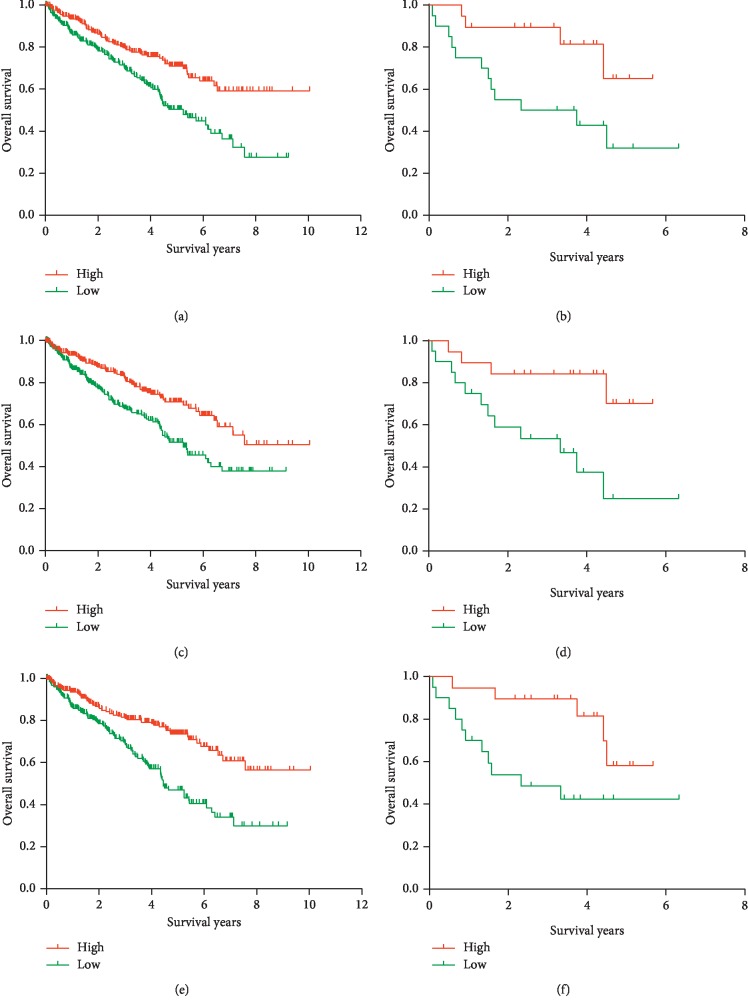
Validation of three downregulated DEGs from the TCGA database in the GEO database: Kaplan–Meier survival curve for (a) HMGCS2 in the TCGA database (*P* < 0.001), (b) HMGCS2 in the GEO database (*P*=0.02), (c) HSD11B2 in the TCGA database (*P* < 0.001), (d) HSD11B2 in the GEO database (*P*=0.01), (e) OGDHL in the TCGA database (*P* < 0.001), and (f) OGDHL in the GEO database (*P*=0.02).

Kaplan–Meier survival analysis was conducted to identify prognostic DEGs with regard to overall survival of ccRCC patients from the TCGA cohort; finally, 43 DEGs were found to be associated with OS in ccRCC. To validate whether the selected DEGs were also predictive of OS in other population, we searched and downloaded a cohort of 39 ccRCC cases with detailed follow-up information from GEO (Supplementary [Supplementary-material supplementary-material-1]). Six DEGs were finally validated to be related to OS, of which three upregulated DEGs including CASP5 (Figure 4(a), *P*=0.005, in the TCGA cohort; Figure 4(b), *P*=0.002, in the GEO validation cohort), HSD11B1 (Figure 4(c), *P*=0.003, in the TCGA cohort; Figure 4(d), *P*=0.05, in the GEO validation cohort), and VSIG4 (Figure 4(e), *P*=0.03, in the TCGA cohort; Figure 4(f), *P*=0.05, in the GEO validation cohort). Three downregulated DEGs included HMGCS2 (Figure 5(a), *P* < 0.001, in the TCGA cohort; Figure 5(b), *P*=0.02, in the GEO validation cohort), HSD11B2 (Figure 5(c), *P* < 0.001, in the TCGA cohort; Figure 5(d), *P*=0.01, in the GEO validation cohort), and OGDHL (Figure 5(e), *P* < 0.001, in the TCGA cohort; Figure 5(f), *P*=0.02, in the GEO validation cohort).

## 4. Discussion

Tumor microenvironment constructed by tumor-associated normal cells during multistep tumorigenesis exerts a significant role in cancer initiation, progression, and drug resistance [12, 13]. Assessing the fraction of microenvironment-associated cell types may illustrate the role of tumor microenvironment and provide perspectives in cancer research. Among the tumor microenvironment, stromal and immune cells constitute the major noncancer proportion. Based on single-sample gene set-enrichment analysis, immune and stromal scores were calculated to predict tumor purity with the level of infiltrating immune and stromal cells by using ESTIMATE [8]. Tumor purity and infiltrated immune/stromal cells are considered to exert a considerable effect on malignancy progression, clinical conditions, and poor prognosis. A tight association between stromal/immune scores and stage as well as prognosis has been demonstrated in glioma [14, 15].

With regard to RCC, it was regarded as a highly vascularized and immunogenic cancer type, harboring two revolutionary therapeutic landscape including anti-angiogenic therapy and immunotherapy [16]. However, heterogeneous tumor microenvironment partly promoted therapeutic resistance and led to unsatisfied prognosis [17]. Chevrier et al. performed immune profiling of samples from 73 ccRCC cases and five healthy controls by mass cytometry and identified 17 tumor-associated macrophage phenotypes, 22 T-cell phenotypes, and a distinct immune composition correlated with progression-free survival [18].

This study firstly evaluated the association between the levels of immune cells or stromal cells and clinical characteristics and found that a higher immune score was related to more advanced status of ccRCC, including T status, N status, M status, tumor grade, as well as the AJCC stage. Moreover, this study demonstrated that a higher immune score was associated with a shorter overall survival, which may be explained by the tight connection between the higher immune score and advanced clinical characteristics; however, the underlying mechanism required more exploration.

Moreover, this study compared the DEGs between higher and lower immune score groups as well as stromal score groups and detected 95 intersective DEGs with 48 upregulated and 47 downregulated genes. The GO and KEGG analysis suggested that the intersected DEGs were involved with immune cell proliferation like T cells, lymphocytes, mononuclear cells and immune response, cytokine-cytokine receptor interaction, as well as NF-kappa B signaling pathway. Among the 95 intersective DEGs, only 43 DEGs were proved to be associated with overall survival based on TCGA follow-up data, and six genes were finally validated for prognostic values in another cohort from the GEO database.

Among the six validated DEGs, VSIG4, HSD11B1, and CASP5 were upregulated in both higher immune score and stromal score groups, and high expression of these genes harbored unfavorable OS compared with low expression groups, suggesting that higher expressions of these genes were predictive of less tumor purity and worse prognosis. On the contrary, the other three downregulated genes including HMGCS2, HSD11B2, and OGDHL in higher immune score and stromal score groups indicated higher expressions of these genes were associated with more tumor purity and prognostic advantages.

VSIG4 was found to promote carcinogenesis by inhibiting cytotoxic T-lymphocyte activation and acted as an independent predictive factor for a shorter progression-free survival and overall survival in high-grade glioma patients [19]. Moreover, macrophage-associated VSIG4 was demonstrated to facilitate lung carcinoma and multiple myeloma development, which provided a promising immunotherapeutic target and prognostic indicator [20, 21]. HSD11B1 and HSD11B2 were two isozymes of 11 beta-hydroxysteroid dehydrogenase and modulate glucocorticoid levels and might influence circulating levels of active and inactive glucocorticoids [22]. HSD11B1 exhibits oncogenic potential in primary imatinib-naïve gastrointestinal stromal tumors driven by HSD11B1 copy-number gain or missense mutations [23]. CASP5, as a member of caspase family, promoted angiogenesis of human microvascular endothelial cells by activating the VEGF-1 signal pathway [24]. CASP5 was also found to be associated with bladder cancer development, especially in the selected cases with smoking exposure [25]. HMGCS2 may function as either an oncogene or a tumor suppressor in various human cancers. HMGCS2 protein expression was significantly reduced in prostate cancer tissues, and low HMGCS2 expression was associated with a high Gleason score, pathological grade, and presence of distant metastasis in prostate cancer [26]. However, in rectal cancer, high expression of HMGCS2 predicted poor disease-free survival, local recurrence-free survival, and metastasis-free survival [27]. OGDHL was found to suppress cervical tumorigenesis via inactivation of the AKT signaling pathway in cervical cancer [28]. A significant high methylation level leading to inactivation of OGDHL was observed in colorectal cancer compared with nontumoral marginal samples and might be considered as a biomarker for colorectal carcinogenesis [29].

With further exploration of public databases and wide application of bioinformatic methods, increasing evidence indicated promising biomarkers and predictive signatures in renal cancer. Based on TCGA and International Cancer Genome Consortium (ICGC) cohorts, DYSF, VNN3, TMED3, and TEK were found and validated to exert as promising biomarkers to predict the prognosis of ccRCC patients [30–33]. A 3-mRNA signature consisting of ERG, RRM2, and EGF was constructed to predict survival in papillary renal cell cancer with satisfactory accuracy [34]. A risk score based on 6 lncRNAs was raised and exhibited superior predictive value for prognosis of ccRCC [35].

There are still some advantages and limitations in this study. The TCGA has enough sample size and comprehensive types of genomic data, providing a relatively reliable basis for bioinformatics analysis. Although the results were further validated in other databases, several DEGs with significant clinical significance in ccRCC are still required to be investigated to determine the underlying molecular mechanism.

## 5. Conclusion

In summary, our study elucidated the close association between immune score and clinical characteristics as well as prognosis in ccRCC. Moreover, six DEGs including CASP5, HSD11B1, VSIG4, HMGCS2, HSD11B2, and OGDHL were explored and validated to exert a predictive value in overall survival of ccRCC.

## Data Availability

Data supporting the findings of this study are available within the article.

## Abbreviations

ccRCC: Clear cell renal cell cancer
DEG: Differentially expressed gene
GO: Gene ontology
BP: Biological processes
MF: Molecular function
KEGG: Kyoto encyclopedia of genes and genomes
PPI: Protein-protein interaction
STRING: Search tool for the retrieval of interacting genes
TCGA: The cancer genome Atlas
GEO: Gene expression omnibus.

## Supplementary Materials

Supplementary Materials Supplementary Figure 1: associations between stromal scores and clinical characteristics or overall survival. (A) Distribution of stromal scores stratified by T status, and box plot indicated a higher stromal score was associated with T3/4 (vs. T1/2, *P*=0.03). (B) Box plot indicated no significant associations between stromal score and N1 (vs. N0, *P*=0.36). (C) Box plot indicated no significant associations between stromal score and M1 (vs. M0, *P*=0.71). (D) Box plot indicated no significant associations between stromal score and G3/4 (vs. G1/2, *P*=0.53). (E) Box plot indicated no significant associations between stromal score and AJCC stage (*P*=0.14). (F) Kaplan–Meier survival curves for overall survival of ccRCC stratified by stromal scores, indicating no significant associations between the low stromal group and high stromal score group (*P*=0.22). Supplementary Table 1: baseline characteristics of TCGA and GEO validation cohorts.
