# Supplementary material for: Deciphering Immune-Associated Genes to Predict Survival in Clear Cell Renal Cell Cancer
Source: Biomed Res Int. 2019 Dec 7;2019:2506843. doi: 10.1155/2019/2506843 (PMC6925759; doi:10.1155/2019/2506843)
Supplement: Supplementary Materials — Supplementary Figure 1: associations between stromal scores and clinical characteristics or overall survival. (A) Distribution of stromal scores stratified by T status, and box plot indicated a higher stromal score was associated with T3/4 (vs. T1/2, P=0.03). (B) Box plot indicated no significant associations between stromal score and N1 (vs. N0, P=0.36). (C) Box plot indicated no significant associations between stromal score and M1 (vs. M0, P=0.71). (D) Box plot indicated no significant associations between stromal score and G3/4 (vs. G1/2, P=0.53). (E) Box plot indicated no significant associations between stromal score and AJCC stage (P=0.14). (F) Kaplan–Meier survival curves for overall survival of ccRCC stratified by stromal scores, indicating no significant associations between the low stromal group and high stromal score group (P=0.22). Supplementary Table 1: baseline characteristics of TCGA and GEO validation cohorts. [file 2506843.f1.docx]

**Supplementary materials**

Supplementary Figure 1. Associations between stromal scores and clinical characteristics or overall survival. (A) Distribution of stromal scores stratified by T status, and box-plot indicated higher stromal score was associated with T3/4 (vs. T1/2, P = 0.03). (B) Box-plot indicated no significant associations between stromal score and N1 (vs. N0, P = 0.36). (C) Box-plot indicated no significant associations between stromal score and M1 (vs. M0, P = 0.71). (D) Box-plot indicated no significant associations between stromal score and G3/4 (vs. G1/2, P = 0.53). (E) Box-plot indicated no significant associations between stromal score and AJCC stage (P = 0.14). (F) Kaplan-Meier survival curves for overall survival of ccRCC stratified by stromal scores, indicating no significant associations between the low stromal group and high stromal score group (P = 0.22)


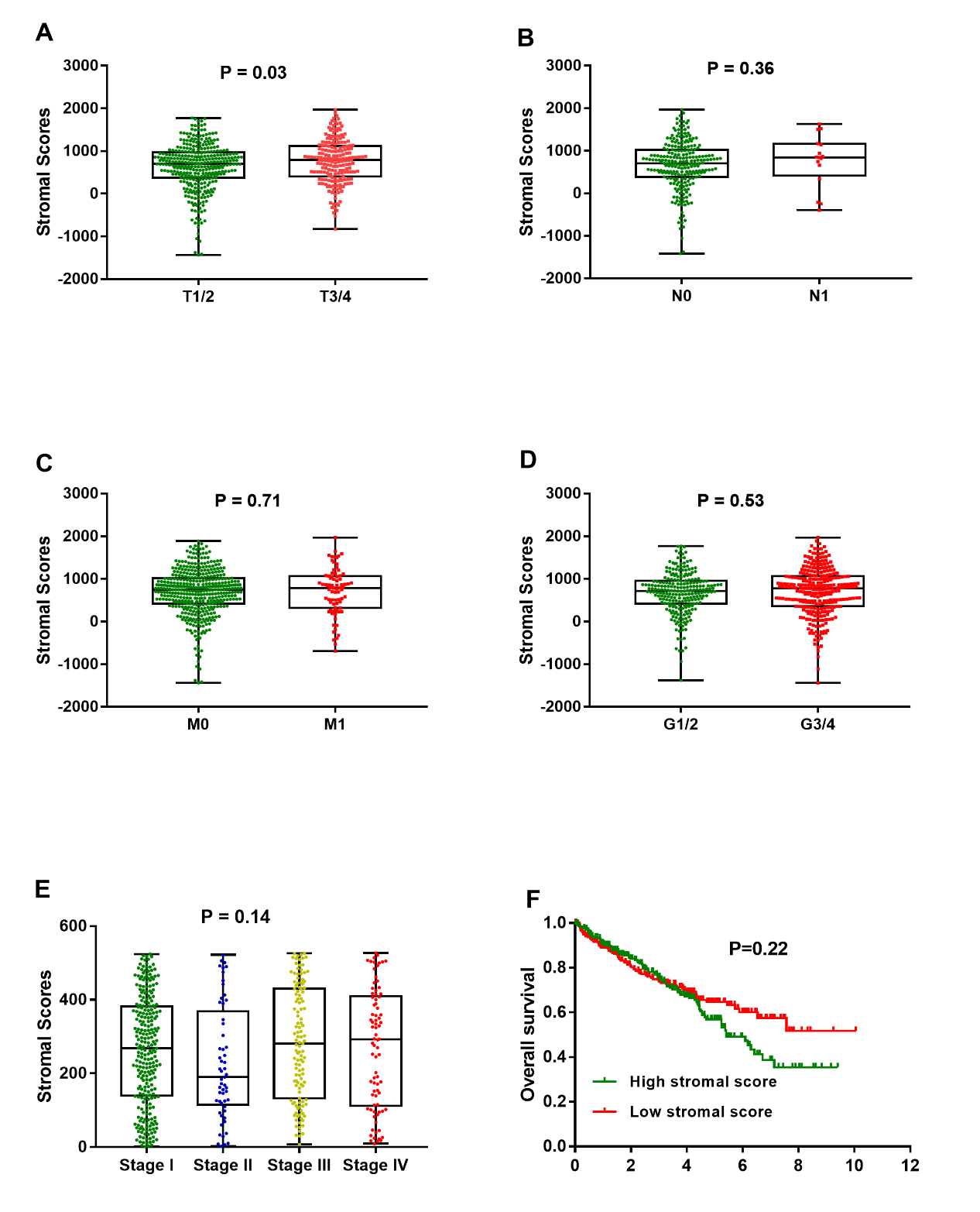


Supplementary Table 1. Baseline characteristics of TCGA and GEO validation cohorts

| **Variants** | | **No. of Patients** | |
| --- | --- | --- | --- |
|  |  | TCGA（n=537） | GEO（n=39） |
| **Age（years）** | ≤60 | 266 | 16 |
|  | 60-70 | 143 | 12 |
|  | ＞70 | 128 | 11 |
| **Gender** | Male | 346 | unknown |
|  | Female | 191 | unknown |
| **AJCC stage** | I | 269 | 11 |
|  | II | 57 | 5 |
|  | III | 125 | 22 |
|  | IV | 83 | 1 |
|  | Unknown | 3 | 0 |
| **Stage T** | T1/T2 | 344 | unknown |
|  | T3/T4 | 193 | unknown |
| **Stage N** | N0 | 240 | 31 |
|  | N1 | 17 | 8 |
|  | Unknown | 280 | 0 |
| **Stage M** | M0 | 426 | 25 |
|  | M1 | 79 | 14 |
|  | Unknown | 32 | 0 |
| **Grade** | G1/2 | 244 | 13 |
|  | G3/4 | 285 | 26 |
|  | Unknown | 8 | 0 |
